# Microcystin-LR Drives Early NAFLD Pathogenesis via Hepatic Cholesterol Accumulation: Dysregulation of *Ldlr* and *Abcg1* Expression Uncoupled from *Srebp2*

**DOI:** 10.3390/toxins18020092

**Published:** 2026-02-11

**Authors:** Hideaki Kawahara, Yoshihito Koto, Yuuka Hitsuda, Koichi Kurata, Keisuke Yoshikiyo, Ayumi Hashiguchi, Hideaki Maseda, Kunihiro Okano, Norio Sugiura, Kazuya Shimizu, Hidehisa Shimizu

**Affiliations:** 1 Graduate School of Life and Environmental Science, Shimane University, 1060 Nishikawatsu-Cho, Matsue 690-8504, Shimane, Japan; 2 Graduate School of Natural Science and Technology, Shimane University, 1060 Nishikawatsu-Cho, Matsue 690-8504, Shimane, Japan; 3 Faculty of Life and Environmental Sciences, Shimane University, 1060 Nishikawatsu-Cho, Matsue 690-8504, Shimane, Japan; 4 Institute of Agricultural and Life Sciences, Academic Assembly, Shimane University, 1060 Nishikawatsu-Cho, Matsue 690-8504, Shimane, Japan; 5 The United Graduate School of Agricultural Sciences, Tottori University, 4-101 Koyama-Minami, Tottori 680-8553, Tottori, Japan; 6 Faculty of Environmental, Life, Natural Science and Technology, Okayama University, 3-1-1, Tsushima-Naka, Kita-ku, Okayama 700-8530, Okayama, Japan; 7 Biomedical Research Institute, National Institute of Advanced Industrial Science and Technology, 1-8-31 Midorigaoka, Osaka 563-8577, Osaka, Japan; 8 Faculty of Bioresource Sciences, Akita Prefectural University, Akita 010-0195, Akita, Japan; 9 Faculty of Life and Environmental Sciences, University of Tsukuba, Tsukuba 305-8572, Ibaraki, Japan; 10 Faculty of Life Sciences, Toyo University, 48-1 Oka, Asaka 351-8510, Saitama, Japan; 11 Bio-Resilience Research Project (BRRP), Toyo University, 48-1 Oka, Asaka 351-8510, Saitama, Japan; 12 Estuary Research Center, Shimane University, 1060 Nishikawatsu-Cho, Matsue 690-8504, Shimane, Japan; 13 Interdisciplinary Center for Science Research, Shimane University, 1060 Nishikawatsu-Cho, Matsue 690-8504, Shimane, Japan

**Keywords:** microcystin-LR, non-alcoholic fatty liver disease (NAFLD), cholesterol homeostasis, low-density lipoprotein receptor (LDLR), ATP-binding cassette transporter G1 (ABCG1), protein phosphatase 2A (PP2A), systems toxicology, metabolic toxicity

## Abstract

Chronic exposure to the cyanotoxin microcystin-LR is an emerging environmental driver of non-alcoholic fatty liver disease (NAFLD); however, the initiating molecular events at sub-lethal, environmentally relevant concentrations remain elusive. Current safety guidelines focus primarily on acute injury, potentially overlooking silent metabolic disruption. The present study investigates the early metabolic toxicity of chronic low-dose microcystin-LR (10 µg/L) in a 7-week rat model, specifically focusing on pre-symptomatic perturbations in lipid homeostasis. By integrating biochemical profiling with multivariate systems toxicology (LASSO and PLS-DA), we identified a specific phenotype of “Silent Hepatic Total Cholesterol Accumulation” (T-CHOL +16%, *p* = 0.01) occurring in the absence of systemic dyslipidemia or overt liver injury. Mechanistic analysis revealed a specific dual failure of cholesterol homeostasis, characterized by the paradoxical upregulation of the influx transporter *Ldlr* (LASSO coef +0.661) and the suppression of the efflux transporter *Abcg1* (PLS1 loading −0.358). Crucially, *Ldlr* upregulation occurred despite the concomitant transcriptional downregulation of *Srebp2* (Spearman ρ = −0.585), indicating a regulatory uncoupling mechanism. We propose that microcystin-LR-induced protein phosphatase 2A (PP2A) inhibition likely drives this uncoupling via a post-transcriptional override—possibly involving ERK/RSK-mediated *Ldlr* mRNA stabilization. Concurrently, this inhibition appears to block LXR-mediated *Abcg1* expression through sustained AMPK hyperactivation resulting from the loss of dephosphorylation. These findings indicate liver-specific cholesterol accumulation as the critical first step of environmental NAFLD pathogenesis, suggesting that current WHO guidelines (1 µg/L) may require re-evaluation regarding metabolic safety. We propose the hepatic *Ldlr*/*Abcg1* ratio as a potential early biomarker for revised risk assessment.

## 1. Introduction

The global proliferation of harmful cyanobacterial blooms (CyanoHABs), driven by accelerating eutrophication and climate change, poses an escalating threat to public health and aquatic ecosystems [1,2]. Among the diverse array of cyanotoxins released, microcystin-LR is the most prevalent and potent congener [3]. Classically, microcystin-LR is recognized as a potent hepatotoxin that specifically targets hepatocytes via organic anion transporting polypeptides (OATPs) [4,5]. Once internalized, it covalently binds to and inhibits protein phosphatases 1 and 2A (PP1 and PP2A), precipitating cytoskeletal collapse, acute hemorrhage, and tumor promotion [4,6,7,8,9]. Consequently, current regulatory frameworks, such as the World Health Organization (WHO) provisional guideline for drinking water (1 µg/L), are primarily calibrated to prevent acute hepatocellular injury [10].

While acute toxicity is well-characterized, an emerging body of epidemiological and experimental evidence suggests a critical blind spot in current safety standards: chronic exposure to low doses of microcystin-LR may function as a metabolic disruptor, contributing to the etiology of non-alcoholic fatty liver disease (NAFLD) [11,12,13,14]. Epidemiological studies have linked CyanoHABs to increased NAFLD incidence [11,12], and recent research using genetic mouse models (e.g., *Lepdb/J*) indicates that chronic PP2A inhibition exacerbates hepatic steatosis and fibrosis even at doses below the No-Observed-Adverse-Effect Level (NOAEL) [15,16,17]. However, a major challenge in risk assessment is the discrepancy between experimental high-dose models and real-world exposure scenarios. The sub-clinical effects of environmentally relevant concentrations—such as peak levels (~10 µg/L) encountered during water treatment compromises or via contaminated irrigation [4,10]—are often overlooked. We hypothesize that such low-dose chronic exposure induces a silent pathology characterized by specific molecular alterations in cholesterol homeostasis that precede overt organ damage or systemic metabolic shifts.

Central to the pathogenesis of NAFLD is the disruption of hepatic cholesterol homeostasis, a process governed by a delicate balance between biosynthesis (mediated by SREBP-2/HMGCR), influx (via LDLR), and efflux (via ABC transporters) [14,18]. Theoretically, the inhibition of PP2A by microcystin-LR creates a regulatory paradox within this network. Canonically, PP2A participates in SREBP-2 activation through the dephosphorylation of upstream regulators, thereby facilitating the nuclear translocation of this master transcriptional regulator [19]. Consequently, its inhibition should logically suppress *Ldlr* expression. However, emerging evidence suggests an alternative, post-transcriptional mechanism: PP2A inhibition activates downstream kinase cascades (e.g., ERK/RSK) that stabilize *Ldlr* mRNA, potentially overriding transcriptional suppression [20,21,22]. Specifically, studies by Rice et al. [19] and Adachi et al. [20] have elucidated this “post-transcriptional override,” demonstrating that phosphatase inhibition can decouple protein levels from transcriptional demand. Determining whether microcystin-LR targets specific nodes within this flux—specifically the uncoupling of influx from transcriptional control—is essential for identifying the molecular first step of its metabolic toxicity.

To address this critical knowledge gap, the present study adopted a systems toxicology framework integrating lipid profiling, gene expression analysis, and multivariate modeling. We exposed rats to 10 µg/L of microcystin-LR via drinking water for 7 weeks. This specific dosage mimics realistic sub-chronic exposure scenarios relevant to human populations in endemic regions. While standard sub-chronic toxicity testing typically employs a 90-day duration (as per FAO/WHO and OECD guidelines), the 7-week duration in this study was specifically selected to capture the “incipient” phase of toxicity—a window we have previously identified in other organs (colon and kidney) as critical for detecting early toxicity markers before the onset of irreversible damage [5,23,24]. Our specific objectives were (1) to characterize the pre-symptomatic metabolic phenotype using Partial Least Squares Discriminant Analysis (PLS-DA); (2) to identify the specific molecular drivers of hepatic cholesterol accumulation (e.g., *Ldlr* and *Abcg1*); and (3) to elucidate the mechanistic disconnect, or “uncoupling,” between transcriptional regulation (SREBP-2) and phenotypic outcome. By defining this early-stage pathology, we aim to provide molecular evidence for refining risk assessment strategies to protect against environmental metabolic diseases.

## 2. Results

### 2.1. Evaluation of Hepatic Morphology, Hepatic Lipid Profiles, and Systemic Metabolic Indices

The present study investigated the first step of metabolic disruption using a rat model exposed non-invasively to an environmentally relevant concentration of microcystin-LR (10 µg/L) via ad libitum drinking water for 7 weeks. In addition to the absence of significant differences in body weight and food intake between the control and microcystin-LR-treated groups as previously reported [5,23], no statistically significant differences were observed in liver weight (both absolute weight and liver-to-body weight ratio) (Table 1). Similarly, regarding major plasma metabolic markers, no significant differences were observed between the groups for glucose, total cholesterol (T-CHOL), triglycerides (TAG), or non-esterified fatty acids (NEFA) (Table 2). This absence of overt organ hypertrophy or systemic dyslipidemia characterizes the “silent” nature of the toxicity at this early exposure stage. In striking contrast to the systemic stability, we observed a specific alteration in the hepatic lipid profile. While hepatic TAG levels showed no statistically significant changes at this time point, hepatic T-CHOL concentrations were significantly elevated (+16%; *p* < 0.05) in the microcystin-LR group compared to controls (Table 3). Collectively, these results suggest that microcystin-LR induces a liver-specific cholesterol overload that occurs independently of, and likely precedes, systemic metabolic alterations. This phenotype represents a critical “incipient” stage of metabolic toxicity.

### 2.2. Alterations in the Expression of Genes Related to Cholesterol Homeostasis

**Figure 1:**
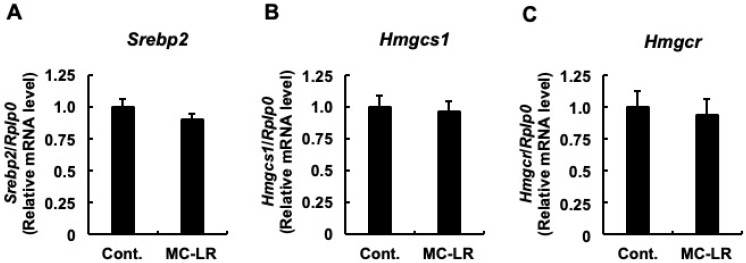
mRNA expression profiles of hepatic cholesterol biosynthesis genes following chronic low-dose microcystin-LR exposure. Relative mRNA expression levels of (**A**) *Srebp2* (gene encoding Sterol regulatory element-binding protein 2), the master transcriptional regulator of cholesterol homeostasis; (**B**) *Hmgcs1* (gene encoding 3-hydroxy-3-methylglutaryl-CoA synthase 1), catalyzing the conversion of acetyl-CoA to HMG-CoA; and (**C**) *Hmgcr* (gene encoding 3-hydroxy-3-methylglutaryl-CoA reductase), the rate-limiting enzyme of cholesterol biosynthesis. Transcript levels were quantified by RT-qPCR and normalized to *Rplp0*. Data represent mean ± standard error (SE) (*n* = 11 per group). No statistically significant differences were observed between the control and microcystin-LR groups (Student’s *t*-test), indicating that de novo synthesis is not the primary driver of lipid accumulation at this stage. Abbreviations: Cont.: Control, MC-LR: microcystin-LR.

**Figure 2:**
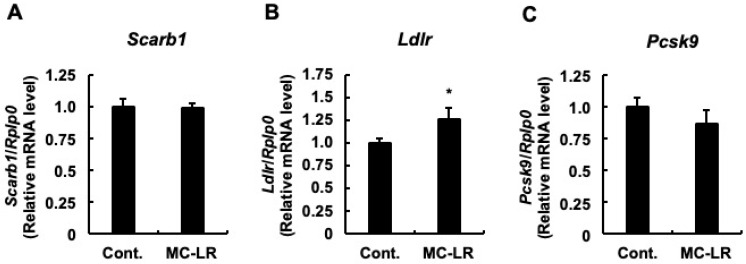
Dysregulation of hepatic cholesterol influx genes and evidence of regulatory uncoupling. Relative mRNA expression levels of (**A**) *Scarb1* (gene encoding scavenger receptor class B member 1), mediating HDL uptake; (**B**) *Ldlr* (gene encoding low-density lipoprotein receptor), the primary receptor for LDL uptake; and (**C**) *Pcsk9* (gene encoding proprotein convertase subtilisin/kexin type 9), a negative regulator of LDLR protein stability. Data represent mean ± SE (*n* = 11 per group). Asterisks indicate significant differences compared to the control group (* *p* < 0.05, Student’s *t*-test). Note the significant upregulation of *Ldlr* (**B**) despite the lack of *Srebp2* induction (Figure 1A), suggesting a regulatory uncoupling mechanism where influx is activated independently of canonical transcriptional control. Abbreviations: Cont.: Control, MC-LR: microcystin-LR.

**Figure 3:**
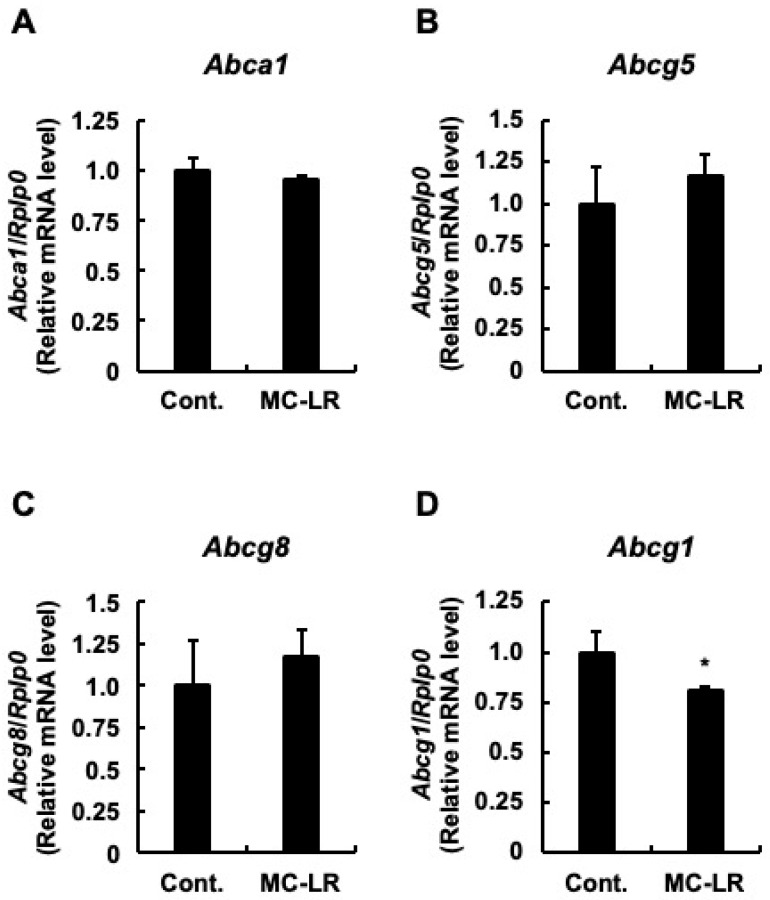
Suppression of hepatic cholesterol efflux transporters. Relative mRNA expression levels of (**A**) *Abca1* (gene encoding ATP-binding cassette transporter A1), mediating efflux to lipid-poor apolipoproteins; (**B**) *Abcg5* (gene encoding ATP-binding cassette transporter G5) and (**C**) *Abcg8* (gene encoding ATP-binding cassette transporter G8), heterodimeric transporters mediating biliary cholesterol secretion; and (**D**) *Abcg1* (gene encoding ATP-binding cassette transporter G1), mediating efflux to HDL particles. Data represent mean ± SE (*n* = 11 per group). Asterisks indicate significant differences compared to the control group (* *p* < 0.05, Student’s *t*-test). The specific downregulation of *Abcg1* (**D**) suggests a compromised efflux pathway, contributing to the dual failure mechanism (enhanced influx/blocked efflux). Abbreviations: Cont.: Control, MC-LR: microcystin-LR.

To elucidate the molecular mechanisms underlying hepatic total cholesterol accumulation, we quantified the mRNA expression levels of genes governing cholesterol synthesis, hepatic influx, and hepatic efflux. First, we examined the synthesis pathway. As shown in Figure 1A–C, no significant differences were observed between the two groups in the mRNA levels of genes involved in cholesterol synthesis. These included *Srebp2*, the representative transcription factor regulating cholesterol homeostasis; *Hmgcs1*, the enzyme responsible for the initial step; and *Hmgcr*, the rate-limiting enzyme. This lack of transcriptional activation suggests that de novo synthesis is not the primary driver of the observed cholesterol accumulation. Next, we analyzed hepatic influx. While *Scarb1* (the HDL receptor) expression remained unchanged (Figure 2A), *Ldlr* mRNA levels—the primary receptor for LDL uptake—were significantly upregulated in the microcystin-LR group compared to controls (Figure 2B; *p* < 0.05). Notably, this upregulation occurred despite the absence of induction in its master regulator, *Srebp2* (Figure 1A), hinting at a regulatory uncoupling or non-canonical mechanism. Although the mean mRNA expression of *Pcsk9*—which promotes LDLR degradation—appeared lower in the microcystin-LR group (consistent with reduced SREBP-2 activity), this difference did not reach statistical significance (Figure 2C). Finally, we investigated hepatic efflux. No significant differences were observed in *Abca1* (Figure 3A) or in *Abcg5* and *Abcg8* (Figure 3B,C). Conversely, the mRNA levels of *Abcg1*, which mediates efflux to HDL, were significantly downregulated in the microcystin-LR group (*p* < 0.05) (Figure 3D). Collectively, these results indicate that the hepatic cholesterol accumulation induced by microcystin-LR is driven by a specific molecular dual failure: the enhanced influx via uncoupled *Ldlr* upregulation and the compromised efflux via *Abcg1* downregulation.

### 2.3. Examination of the Oxysterol Generation Pathway and Compensatory Responses

**Figure 4:**
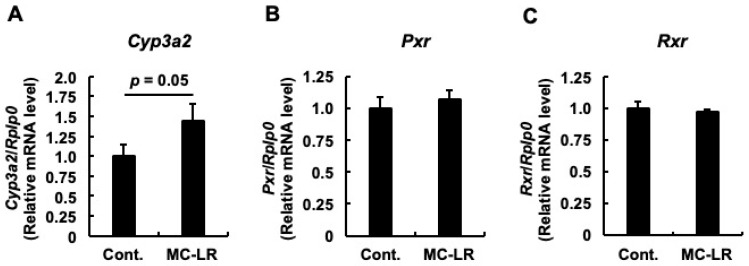
Activation of the oxysterol generation pathway as a potential compensatory response. Relative mRNA expression levels of (**A**) *Cyp3a2* (gene encoding cytochrome P450 3A2), involved in cholesterol catabolism to oxysterols; (**B**) *Pxr* (gene encoding pregnane X receptor); and (**C**) *Rxr* (gene encoding retinoid X receptor). Data represent mean ± SE (*n* = 11 per group). While univariate analysis showed a marginally significant increase in *Cyp3a2* (*p* = 0.05, Student’s *t*-test), this gene was consistently identified as a key driver in subsequent multivariate analyses. This upregulation occurred independently of PXR/RXR induction, suggesting a metabolic feedback response to the hepatic cholesterol overload. Abbreviations: Cont.: Control, MC-LR: microcystin-LR.

Previous studies have reported that microcystin-LR intake induces fatty liver phenotypes in zebrafish and mice [13,24]. Given that hepatic steatosis is often accompanied by elevated oxysterols due to cholesterol overload [25], we investigated the mRNA expression of *Cyp3a2* (the rat homolog of human *CYP3A4*), which is responsible for oxysterol generation. As shown in Figure 4A, *Cyp3a2* mRNA levels exhibited an increasing trend in the microcystin-LR group compared to the control. Although this difference reached borderline significance in the univariate analysis (*p* = 0.05), its relevance to the overall toxicological profile was robustly substantiated by subsequent multivariate analyses (see Section 2.5), where it was identified as a key driver of the toxicity model. We subsequently examined the upstream regulators of *Cyp3a2*. No significant differences were observed in the mRNA levels of the transcription factors *Pxr* and *Rxr* (Figure 4B,C). These findings suggest that the upregulation of *Cyp3a2* occurred independently of increased *Pxr*/*Rxr* expression, possibly reflecting a compensatory metabolic feedback response to generate oxysterols in reaction to the hepatic cholesterol accumulation identified in Section 2.1.

### 2.4. Examination of Transcriptional Changes Related to Triglyceride Metabolism

**Figure 5:**
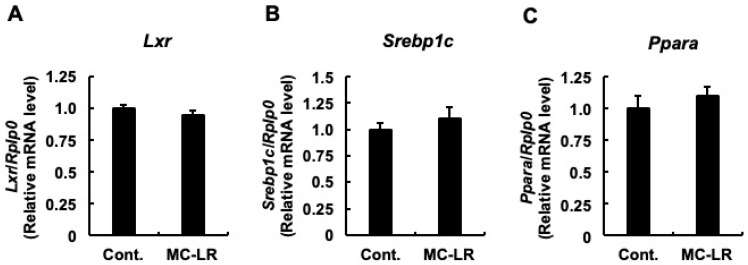
Transcriptional status of triglyceride metabolism regulators. Relative mRNA expression levels of (**A**) *Lxr* (gene encoding liver X receptor); (**B**) *Srebp1c* (gene encoding sterol regulatory element-binding protein 1c), the master regulator of lipogenesis; and (**C**) *Ppara* (gene encoding peroxisome proliferator-activated receptor alpha), regulating β-oxidation. Data represent mean ± SE (*n* = 11 per group). No statistically significant differences were observed (Student’s *t*-test), consistent with the unaltered hepatic triglyceride levels. This indicates that the lipogenic axis remains relatively intact at this early stage of toxicity (pathological transition phase). Abbreviations: Cont.: Control, MC-LR: microcystin-LR.

Given the findings in Figure 4 suggesting early oxysterol accumulation (which typically activates LXR), we next investigated the downstream transcriptional regulators of fatty acid metabolism to determine if the toxicity had spread to lipogenesis. We focused on the mRNA levels of *Lxr* (the oxysterol receptor), its target gene *Srebp1c* (master regulator of fatty acid synthesis), and *Ppara* (promoter of β-oxidation). Regarding *Lxr*, no significant difference was observed in mRNA levels between the two groups (Figure 5A). Similarly, for *Srebp1c* and *Ppara*, although mean values were elevated in the microcystin-LR group compared to controls, these differences did not reach statistical significance (Figure 5B,C). However, when considered alongside the upward trend in plasma TAG levels (Table 3), these combined results hint at the initiation of hepatic TAG accumulation. Although differences did not reach statistical significance at this 7-week time point, the observed trends suggest a potential time-dependent activation of the LXR/SREBP-1c axis that may become overt with continued exposure. This interpretation aligns with the concept of a pathological transition phase, where cholesterol dysregulation precedes massive triglyceride accumulation.

### 2.5. Integration of Toxic Effects via Multivariate Analysis and Quantitative Evaluation of Molecular Mechanisms

#### 2.5.1. Robust Group Separation via PLS-DA Reveals Silent Toxicity

**Figure 6:**
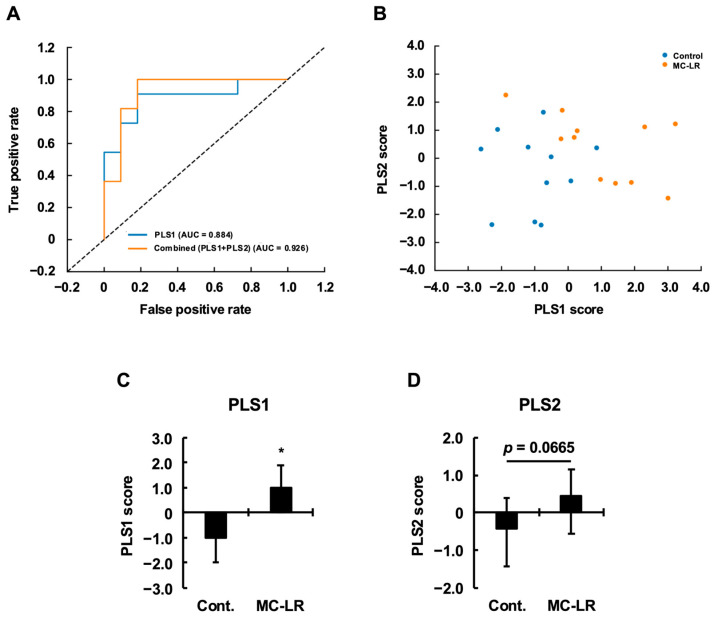
Multivariate statistical evaluation of metabolic profiles using PLS-DA. (**A**) Receiver Operating Characteristic (ROC) curves for the PLS1 classifier (univariate) and the combined PLS1 + PLS2 classifier (via logistic regression). The high Area Under the Curve (AUC) values (0.884 and 0.926, respectively) indicate robust discriminative ability. The diagonal dashed line represents the chance level. (**B**) PLS-DA score plot (PLS1 vs. PLS2) constructed using all measured variables, showing distinct clustering between control (blue) and microcystin-LR (orange) groups. (**C**) Statistical comparison of PLS1 scores and (**D**) PLS2 scores between groups. Data represent mean ± SE (*n* = 11 per group). Asterisks indicate significant differences compared to the control group (* *p* < 0.05, Student’s *t*-test). In (**D**), the *p*-value indicates a marginal trend (*p* = 0.0665). Note that the significant separation along PLS1 reflects the toxicity-driven metabolic fingerprint, whereas PLS2 captures independent physiological lipid variation. Abbreviations: PLS-DA: Partial Least Squares Discriminant Analysis, ROC: Receiver Operating Characteristic, AUC: Area Under the Curve, Cont.: Control, MC-LR: microcystin-LR.

To evaluate the impact of microcystin-LR beyond univariate effects, we performed Partial Least Squares Discriminant Analysis (PLS-DA) using all measured variables (*n* = 22 samples). Receiver operating characteristic (ROC) analysis demonstrated strong discriminative performance on the full dataset, with AUCs of 0.884 for PLS1 and 0.926 for the combined classifier integrating PLS1 and PLS2 (Figure 6A). To rigorously assess generalizability given the modest sample size, we conducted both stratified cross-validation and nonparametric bootstrap resampling. Five-fold stratified cross-validation yielded mean ± SE AUC values of 0.550 ± 0.123 (PLS1) and 0.683 ± 0.093 (combined classifier), with substantial variability likely attributable to limited sample size per fold (Table S1). In contrast, 1000-iteration stratified bootstrap resampling provided robust 95% confidence intervals, ranging from 0.702–1.000 (PLS1) to 0.777–1.000 (combined), supporting the stability of the full-dataset separation. Consistent with these findings, the PLS-DA score plot (Figure 6B) revealed clear group separation: control samples (blue) were distributed predominantly in the negative PLS1 region, whereas microcystin-LR-exposed samples (orange) shifted positively. Statistical comparison of PLS1 confirmed a substantial effect (*t* = 3.568, *p* = 0.0011; Cohen’s *d* = 1.596, *η*^2^ = 0.389, Figure 6C; Table 4). PLS2 showed only marginal group differences (*t* = 1.567, *p* = 0.0665; Cohen’s *d* = 0.701, *η*^2^ = 0.109, Figure 6D; Table 4). Multivariate testing further validated these results: MANOVA indicated significant overall group differences (*p* = 0.0014 across Wilks’ lambda, Pillai’s trace, Hotelling–Lawley trace, and Roy’s largest root; Table 5). Taken together, these findings demonstrate that subtle univariate alterations (Section 2.1, Section 2.2, Section 2.3 and Section 2.4) integrate into a distinct, robust multivariate toxicological fingerprint, revealing microcystin-LR-induced silent toxicity that becomes apparent only when evaluated at the systems level.

#### 2.5.2. Loading Analysis of the PLS1 Axis and Identification of Toxicity Drivers

To interpret the biological significance of the toxicity-specific PLS1 axis, we evaluated the loading weights (contribution) of each variable (Table 6). The PLS1 axis was robustly driven by variables with positive loadings, most notably Liver T-CHOL (Loading = +0.453) and *Ldlr* (Loading = +0.377). Conversely, variables with negative loadings included *Abcg1* (Loading = −0.358), *Lxr* (Loading = −0.291), and *Srebp2* (Loading = −0.261). This distinctive loading pattern—where *Ldlr* and *Abcg1* pull in opposite directions—statistically corroborates the dual failure mechanism (enhanced influx vs. compromised efflux) proposed in Section 2.2. Furthermore, the opposing signs of *Ldlr* (positive) and *Srebp2* (negative) loadings provide quantitative evidence for the uncoupling phenomenon, confirming that *Ldlr* upregulation occurs independently of its canonical transcriptional master regulator. Additionally, the positive loading of *Cyp3a2* (Loading = +0.340) aligns with the compensatory response hypothesis. Consistent with these loadings, Variable Importance in Projection (VIP) analysis identified Liver T-CHOL (VIP = 1.879), *Ldlr* (VIP = 1.591), and *Abcg1* (VIP = 1.505) as having the highest contributions (Table 7). These results quantitatively suggest *Ldlr* and *Abcg1* as the primary molecular drivers and potential early biomarkers of microcystin-LR metabolic toxicity.

#### 2.5.3. Quantitative Verification of Driving Factors (PLS1 Axis and Liver T-CHOL)

To quantitatively validate the molecular mechanisms underlying the toxicity-specific PLS1 axis and Liver T-CHOL accumulation, we performed LASSO regression analysis and Spearman correlation analysis (Table 8, Table 9, Table 10 and Table 11). First, we analyzed the drivers of the PLS1 axis. LASSO regression using the PLS1 score as the target variable revealed that variations in PLS1 were robustly predicted by positive coefficients for Liver T-CHOL (+0.485), *Cyp3a2* (+0.375), and *Ldlr* (+0.354), contrasted by negative coefficients for *Srebp2* (−0.451), *Lxr* (−0.383), and *Abcg1* (−0.240) (Table 8). In particular, *Ldlr* exhibited a very strong positive correlation with the PLS1 score (Spearman ρ = +0.692, *p* = 0.00035). Crucially, *Srebp2* and *Pcsk9* showed significant negative correlations with the toxicity axis (*Srebp2*: Spearman ρ = −0.585, *p* = 0.0042; *Pcsk9*: Spearman ρ = −0.579, *p* = 0.0047) (Table 9). This statistical inversion provides strong quantitative evidence for the uncoupling between toxicity progression and the canonical SREBP-2 transcriptional pathway. Next, we analyzed the predictors of Liver T-CHOL accumulation. LASSO regression with Liver T-CHOL as the target variable identified *Ldlr* (+0.661) as the most powerful positive predictor (Table 10). Furthermore, a significant positive correlation was confirmed between Liver T-CHOL and *Ldlr* (Spearman ρ = +0.535, *p* = 0.010). Regarding *Cyp3a2*, while the univariate group comparison was borderline (*p* = 0.05), Spearman correlation analysis confirmed a statistically significant positive association with Liver T-CHOL (Spearman ρ = +0.426, *p* = 0.048). This is consistent with the multivariate support (PLS1 loading +0.340) and suggests a compensatory metabolic response to cholesterol accumulation (Table 11). These results directly corroborate that excessive cholesterol uptake driven by uncoupled *Ldlr* upregulation is the primary cause of hepatic T-CHOL accumulation.

#### 2.5.4. Evaluation of the PLS2 Axis and Independence of TAG Metabolism

The PLS2 axis captures sources of biological variation that are orthogonal (independent) to the toxicological effects captured by PLS1. To characterize the biological identity of this axis, we analyzed loading weights, LASSO regression coefficients, and Spearman correlations (Table 12, Table 13 and Table 14). The PLS2 axis was primarily driven by general markers of lipid synthesis and metabolism. Loading analysis (Table 12) and LASSO regression (Table 13) consistently identified *Srebp1c*, Plasma T-CHOL, and Plasma TAG as the top positive drivers. Specifically, LASSO coefficients were +0.584 for *Srebp1c*, +0.423 for Plasma T-CHOL, and +0.281 for Plasma TAG. Spearman analysis further confirmed extremely strong positive correlations between the PLS2 score and these markers (Plasma TAG: Spearman ρ = +0.745, *p* < 0.0001; *Srebp1c*: Spearman ρ = +0.685, *p* = 0.0004) (Table 14). To further validate the regulatory mechanism of this axis, we investigated the predictors of Plasma TAG using LASSO regression and Spearman correlation analyses (Table 15 and Table 16). Plasma TAG exhibited a significant positive correlation with *Srebp1c* (Spearman ρ = +0.673, *p* = 0.0005) (Table 16). Most notably, LASSO analysis identified *Srebp1c* as the predominant predictor of Plasma TAG variations, with a remarkably high coefficient (+28.028) compared to other variables (Table 15). These results quantitatively confirm that the PLS2 axis represents canonical physiological lipid metabolism predominantly under the control of *Srebp1c*. Crucially, the preservation of this canonical mRNA-phenotype correlation (*Srebp1c* → TAG) on the PLS2 axis stands in stark contrast to the uncoupling (*Srebp2* ≠ *Ldlr*) observed on the toxicity axis (PLS1). This dissociation further validates that microcystin-LR toxicity specifically targets the cholesterol flux pathway while leaving the SREBP-1c lipogenic axis largely intact at this early stage.

## 3. Discussion

### 3.1. Uncovering the Silent Pathology of Environmental Microcystin-LR Exposure

The primary objective of the present study was to address a critical blind spot in environmental risk assessment: the sub-clinical metabolic toxicity induced by chronic exposure to environmentally relevant concentrations of microcystin-LR (10 µg/L). By employing a systems toxicology framework, we successfully characterized the first step of pathogenesis that precedes overt organ injury [13,26]. Our results reveal that while systemic metabolic indices and hepatic morphology remained deceptively stable, the liver was undergoing a profound metabolic disruption. Specifically, multivariate modeling (PLS-DA) identified a silent but robust toxicity characterized by significant hepatic cholesterol accumulation (Effect size; Cohen’s *d* = 1.596), which was statistically segregated from physiological lipid fluctuations (PLS2 axis). Mechanistically, LASSO and correlation analyses pinpointed the driver of this pathology as a specific dual failure of flux control: the enhanced influx via uncoupled *Ldlr* upregulation and the concurrent compromise of efflux via *Abcg1* downregulation. These findings indicate that microcystin-LR functions as a chronic stealth metabolic disruptor, initiating liver-specific cholesterol overload well before the detection of conventional markers of toxicity.

### 3.2. Redefining Toxicity: The Silent Metabolic Phase vs. Acute Injury

The identification of hepatic cholesterol accumulation in the absence of systemic dyslipidemia or organ hypertrophy challenges the conventional understanding of microcystin-LR toxicity. Classically, microcystin-LR is characterized as a potent hepatotoxin causing cytoskeletal collapse, intrahepatic hemorrhage, and rapid hepatocellular necrosis [6,27]. Current risk assessments, including the WHO guideline, rely heavily on these acute endpoints [10]. In contrast, our systems toxicology approach using an environmentally relevant concentration (10 µg/L) revealed a distinct silent pathology. While plasma markers (TAG, T-CHOL, Glucose) remained within physiological ranges—mimicking a healthy clinical profile—the liver had already initiated a pathological trajectory characterized by specific cholesterol overload. This discrepancy suggests that metabolic disruption acts as the “first step” of toxicity, occurring at exposure levels far below those required to trigger overt architectural damage. This finding provides a critical expansion of the conceptual framework for environmental hepatotoxins: chronic low-dose exposure does not merely cause milder acute injury, but rather induces a qualitatively different metabolic syndrome-like phenotype. Recognizing this silent phase is crucial for early intervention, as reliance on standard serum biomarkers would fail to detect this incipient toxicity until significant liver pathology (e.g., NASH/Fibrosis) has developed.

### 3.3. The Dual Failure Mechanism: Uncoupling of Influx and Suppression of Efflux

Our systems toxicology analysis strongly suggests that the dysregulation of *Ldlr* and *Abcg1* represents the core molecular driver of the observed toxicity (PLS1 axis). This points to a specific dual failure of cholesterol flux control: enhanced uptake combined with compromised excretion.

#### 3.3.1. The Influx Paradox: Uncoupling of Ldlr from SREBP-2

The identification of *Ldlr* as the strongest positive predictor of hepatic cholesterol accumulation (LASSO Coeff. +0.662) presents a fascinating regulatory paradox. Canonically, *Ldlr* transcription is tightly governed by SREBP-2, which is activated when cellular cholesterol is low [19]. However, our data revealed a significant negative correlation between *Srebp2* and the toxicity axis (Table 9), indicating that *Ldlr* was upregulated despite the suppression of its master transcriptional regulator. We propose that this uncoupling is likely driven by the specific molecular action of microcystin-LR: the inhibition of PP2A. Previous mechanistic studies have established that PP2A inhibition activates downstream kinase cascades (e.g., ERK/RSK) that target the 3′-untranslated region (UTR) of *Ldlr* mRNA, preventing its degradation and extending its half-life [20,21,22]. Thus, microcystin-LR likely induces a false signal of cholesterol starvation or mechanically stabilizes *Ldlr* mRNA via post-transcriptional mechanisms, forcing the liver to import excessive cholesterol even when intracellular levels are already dangerously high. This putative post-transcriptional mechanism may explain why the phenotype (cholesterol accumulation) appears to contradict the canonical transcriptional status (SREBP-2 suppression).

#### 3.3.2. The Efflux Failure: Abcg1 Downregulation

Compounding this enhanced influx is the significant downregulation of *Abcg1* (Figure 3D), the transporter responsible for cholesterol efflux to HDL. This creates a functional imbalance in which cholesterol influx exceeds efflux capacity. The suppression of *Abcg1* is likely secondary to the inhibition of the LXR signaling axis (suggested by the negative PLS1 loading of *Lxr*), possibly due to the disruption of oxysterol signaling or direct crosstalk with PP2A inhibition pathways [26,28]. Together, this Dual Failure—non-canonical influx driven by mRNA stabilization and compromised efflux driven by transporter suppression—constitutes the likely mechanistic basis of the first step of microcystin-LR-induced NAFLD.

### 3.4. The Mechanism of “Uncoupling”: Post-Transcriptional Stabilization of Ldlr

The defining molecular feature of the observed toxicity is the paradoxical upregulation of *Ldlr* despite the suppression of its master transcriptional regulator, *Srebp2*. This uncoupling—quantitatively confirmed by the inverse correlation in our LASSO/Spearman analyses (Spearman ρ = −0.585)—defies the canonical model where *Ldlr* transcription is strictly driven by SREBP-2 activation in response to low cellular cholesterol [19]. We propose that this anomaly represents a specific molecular override driven by the primary pharmacological action of microcystin-LR: the inhibition of PP2A.

#### The Disinhibition Hypothesis

Canonically, PP2A is required to activate SREBP-2; thus, its inhibition should logically suppress *Ldlr* expression. However, emerging evidence indicates that PP2A also acts as a brake on the ERK/RSK signaling cascade, which regulates mRNA stability [20,21,22]. Under physiological conditions, RNA-binding proteins (RBPs) such as ZFP36L1/L2 promote the rapid decay of *Ldlr* mRNA. The inhibition of PP2A by microcystin-LR releases the brake on ERK/RSK signaling, leading to the phosphorylation and inactivation of these RBPs. This results in a disinhibition mechanism: the suppression of the decay machinery stabilizes *Ldlr* mRNA, extending its half-life and causing it to accumulate intracellularly independently of transcriptional demand. This post-transcriptional mechanism explains why the liver continues to import cholesterol (high *Ldlr*) even when the transcriptional feedback loop is attempting to shut it down (low *Srebp2*). This mechanistic insight validates our mRNA-centric findings and suggests that microcystin-LR toxicity functions by hijacking the stability control of key metabolic receptors.

### 3.5. The Efflux Blockade: A “Molecular Trap” Driven by the PP2A-AMPK-LXR Axis

Compounding the enhanced influx is the significant downregulation of the efflux transporter *Abcg1* (Figure 3D). This imbalance creates a molecular context wherein cholesterol enters the hepatocyte via stabilized *Ldlr* mRNA but cannot exit. Our multivariate analysis identified *Abcg1* as a strong negative driver of the toxicity axis (Loading = −0.358), confirming that the loss of efflux capacity is an independent and critical component of the dual failure. The suppression of *Abcg1* is particularly striking because intracellular cholesterol accumulation should theoretically trigger a defensive upregulation of efflux transporters via the LXR pathway. The fact that *Abcg1* is suppressed suggests that this canonical defense mechanism is being overridden by a dominant inhibitory signal. We propose that this override is mediated by the PP2A-AMPK axis. PP2A acts as a physiological phosphatase that inactivates AMP-activated protein kinase (AMPK) [29,30]. Consequently, microcystin-LR-induced PP2A inhibition leads to sustained AMPK phosphorylation (hyperactivation). Crucially, activated AMPK is known to directly inhibit LXR transcriptional activity regardless of ligand presence [26]. Thus, the microcystin-LR-induced hyperactivation of AMPK effectively shuts down the LXR-driven efflux pathway, likely contributing to the accumulation.

### 3.6. Failed Compensatory Responses and the “Incipient” Nature of Toxicity

Despite this blockade, the hepatocyte attempts to mount a metabolic defense. The upregulation of *Cyp3a2*—identified as a key feature in our multivariate model (LASSO Coeff. +0.375; Spearman ρ = +0.426)—likely reflects a compensatory catabolic response to detoxify accumulating cholesterol by converting it into oxysterols (LXR ligands) [14,18]. Under physiological conditions, these oxysterols act as potent endogenous ligands for LXR to trigger the expression of efflux transporters [31]. However, this effort represents a failed rescue. While *Cyp3a2* successfully generates oxysterols (the signal), the downstream effector (*Abcg1*) remains blocked by the AMPK-LXR interference described above. This signal-response disconnection further exacerbates the accumulation. Importantly, this specific molecular profile distinguishes our findings as the “first step” of pathogenesis. In advanced human NAFLD/NASH, CYP3A4 activity is typically reduced due to severe inflammation and fibrosis [32,33]. In contrast, the upregulation of *Cyp3a2* observed here indicates that the liver is still in a reactive, pre-symptomatic phase. Furthermore, the lack of significant *Srebp1c* or *Ppara* dysregulation (Section 2.4) confirms that the toxicity has not yet progressed to generalized steatosis (fatty acid accumulation). Therefore, we conclude that microcystin-LR exposure induces a specific, incipient metabolic toxicity restricted to cholesterol flux disruption, occurring well before the onset of the broad metabolic collapse seen in advanced disease.

### 3.7. Exclusion of De Novo Synthesis and Implications for Therapeutic Strategies

Our results strongly suggest against enhanced biosynthesis as a driver of the observed cholesterol accumulation. The mRNA levels of the rate-limiting enzyme *Hmgcr* remained unchanged (Figure 1C), and the *Pcsk9* trend was suppressive, indicating that the SREBP-2 pathway is responding normally to the cholesterol overload by shutting down.

#### 3.7.1. Post-Translational Inactivation of HMGCR

Critically, the pharmacological action of microcystin-LR reinforces this synthesis shutdown hypothesis at the post-translational level. Canonically, HMGCR enzymatic activity is strictly regulated by phosphorylation: it is inactivated by AMPK and activated (dephosphorylated) by PP2A [19,34,35]. Therefore, microcystin-LR-induced PP2A inhibition exerts a double negative effect on synthesis: (1) it prevents the direct dephosphorylation (activation) of HMGCR, and (2) it sustains the activity of AMPK (the inhibitor of HMGCR) [29,33]. This theoretical framework strongly suggests that hepatic cholesterol synthesis is functionally suppressed at both the transcriptional and post-translational levels.

#### 3.7.2. Clinical Implications: Environmental vs. Lifestyle NAFLD

This distinction—that toxicity is driven by influx (*Ldlr*) rather than synthesis—has profound clinical implications. Unlike lifestyle-induced NAFLD, where de novo lipogenesis is often elevated [14,18], microcystin-LR-induced metabolic toxicity may be refractory to standard HMGCR inhibitors (statins). Instead, therapeutic strategies targeting cholesterol absorption (e.g., Ezetimibe) [14] or promoting efflux (e.g., LXR agonists) [18] may prove more effective. This etiological distinction emphasizes the need to consider environmental exposure history when determining treatment strategies for idiopathic NAFLD.

### 3.8. Pathway Specificity: Differential Dependence of SREBP Isoforms on PP2A

A key question arising from our silent pathology finding is the exquisite specificity of the toxicity: why is cholesterol homeostasis severely disrupted while triglyceride metabolism (SREBP-1c pathway) remains relatively stable? Our multivariate analysis (PLS2 axis) confirmed that SREBP-1c maintains a canonical correlation with plasma TAG, distinct from the toxicity axis. This specificity can be rationalized by the differential regulatory dependence of SREBP isoforms on PP2A activity. While PP2A is established as an essential activator for SREBP-2 (cholesterol) [19], its role in SREBP-1c (fatty acid) regulation appears less critical. Studies utilizing siRNA knockdown of the PP2A catalytic subunit (*Ppp2cα*) have demonstrated that while SREBP-2 targets are significantly affected, SREBP-1c target genes (e.g., *Fas* and *Acc1*) remain largely unresponsive [19,36]. This suggests that the LXR/SREBP-1c lipogenic axis possesses a regulatory redundancy or independence that shields it from the immediate effects of PP2A inhibition. Consequently, microcystin-LR toxicity manifests primarily as a cholesterol-specific disorder in the early phase, driven by the Dual Failure (*Ldlr*/*Abcg1*) and Uncoupling, while the lipogenic machinery remains intact until secondary stress signals (e.g., massive oxysterol accumulation) eventually force a transition to steatosis. This mechanistic insight reinforces our conclusion that we have captured the precise first step of the metabolic cascade.

### 3.9. Integrative Model: How PP2A Inhibition Orchestrates the Silent Cholesterol Overload

In summary, the specific hepatic T-CHOL accumulation observed in this study is not a random metabolic disturbance but the specific outcome of microcystin-LR-induced PP2A inhibition acting on regulatory nodes. We propose an integrative mechanistic model characterized by a “Molecular Override” and “Pathway Specificity”:

1. The Cholesterol Trap (The Override): For the cholesterol pathway, PP2A inhibition disrupts the canonical logic. While the transcriptional master regulator (SREBP-2) is appropriately suppressed by the rising cholesterol levels (negative feedback), microcystin-LR short-circuits this Control. It activates a post-transcriptional mechanism (likely via ERK/RSK) that stabilizes *Ldlr* mRNA, forcing continued influx. Simultaneously, it creates an efflux blockade via the AMPK-mediated suppression of *Abcg1*. This Dual Failure traps cholesterol within the hepatocyte.
2. The Lipogenic Stability (The Specificity): Conversely, the lipogenic pathway (SREBP-1c) remains relatively stable in this incipient phase. This is likely due to the differential dependence on PP2A, where the SREBP-1c machinery is less sensitive to the immediate loss of phosphatase activity compared to the strictly regulated SREBP-2/HMGCR axis.

This imbalance—specifically, the uncoupled hyper-activation of influx (*Ldlr*) versus the maintained but non-compensatory lipogenesis—constitutes the likely molecular mechanism driving the specific pathology. By defining this molecular profile, we have successfully mapped the first step of environmental metabolic toxicity, occurring silently before the onset of systemic dyslipidemia or overt liver injury.

### 3.10. The Pathological Transition: From Silent Toxicity to Overt NAFLD

Finally, our systems toxicology approach allows us to place the observed toxicity on a temporal timeline of NAFLD progression. The accumulation of free cholesterol is known to be cytotoxic, secondarily inducing ER stress and oxidative stress, which triggers the transition to NASH [37]. We propose that our study captures the critical “Pathological Transition Phase” mediated by an “Oxysterol Switch.” As noted in Section 3.6, the liver attempts to catabolize the excess cholesterol via *Cyp3a2* (upregulated trend), generating oxysterols such as 4β-hydroxycholesterol. While these oxysterols fail to rescue efflux (due to the AMPK blockade), they act as potent ligands for LXR to promote lipogenesis via *Srebp1c* [28]. Our data supports this sequential model: while the cholesterol phenotype is fully established (PLS1 axis), the lipogenic markers (*Lxr*, *Srebp1c*, plasma TAG) show only increasing trends without statistical significance (Section 2.4). This interpretation is robustly corroborated by our multivariate analysis structure:

1. PLS1 (The First Step): Driven by *Ldlr* and *Abcg1*, representing the established specific toxicity of cholesterol disruption.
2. PLS2 (The Second Step): Driven by *Srebp1c* and TAG, representing the initiating physiological response.

Thus, we conclude that chronic exposure to low-dose microcystin-LR creates a silent cholesterol overload first, which then acts as a metabolic trigger to forcibly activate the LXR/SREBP-1c axis, eventually leading to the overt fatty liver (TAG accumulation) observed in longer-term or higher-dose studies. Identifying this very first step is critical, as it precedes the irreversible inflammatory cascades (e.g., NOX2-miR-21 axis) associated with advanced fibrosis [38].

### 3.11. Strengths and Limitations: The Triangulation of Evidence

A primary limitation of this study is that our analysis was restricted to mRNA expression profiling, and we did not directly quantify LDLR protein levels or assess kinetic uptake activity. However, the validity of our conclusion regarding the functional upregulation of LDLR is robustly supported by the triangulation of three independent lines of evidence: “Phenotypic Anchoring,” “Mechanistic Congruence,” and “Statistical Bridging.”

1. Phenotypic Anchoring (Deductive Logic): We observed a highly significant accumulation of hepatic total cholesterol (Table 2) as a functional endpoint. Crucially, the cholesterol biosynthesis pathway, including the rate-limiting enzyme *Hmgcr* and the master regulator *Srebp2*, remained completely unchanged or suppressed (Figure 1) [19,34]. By deductive elimination, in the absence of increased synthesis, the observed accumulation can logically only be attributed to enhanced uptake. Therefore, the upregulation of *Ldlr* mRNA serves as the only plausible molecular explanation for the phenotype.
2. Mechanistic Congruence (Biological Plausibility): Our findings align consistently with the established mode of action of microcystin-LR. Previous studies have demonstrated that PP2A inhibition specifically promotes LDLR accumulation by stabilizing its mRNA via the ERK-RSK pathway [20,21,22]. Our data—showing *Ldlr* upregulation despite *Srebp2* suppression—is the specific molecular signature of this post-transcriptional mechanism [19]. Furthermore, the potential for microcystins to modulate cellular receptor levels through PP2A inhibition is supported by early structure–function studies [39]. Thus, the mRNA change in this context is not a transient fluctuation but a direct reflection of the stabilizing toxicity.
3. Statistical Bridging (Quantitative Robustness): Our multivariate analysis bridged the gap between gene expression and metabolic outcome. LASSO regression identified *Ldlr* as the strongest positive predictor of liver T-CHOL levels (Coefficient = +0.661), with a significant correlation (Spearman ρ = +0.535). This statistical strength indicates that *Ldlr* variation is likely bound to the pathological outcome.

Taken together, while direct protein quantification was not performed, this convergence of phenotypic, mechanistic, and statistical evidence provides a sufficient and robust rationale to conclude that microcystin-LR drives NAFLD pathogenesis via LDLR-mediated cholesterol influx.

### 3.12. Future Directions and Public Health Significance

Since these results are based on a rat model, extrapolating the findings to human populations requires careful validation. Future research should prioritize two key areas: (1) epidemiological studies in microcystin-LR-exposed regions to verify the association between silent cholesterol abnormalities and early-stage NAFLD, and (2) in vitro studies using advanced human liver models [40,41] to confirm whether the specific “Dual Failure” (*Ldlr*/*Abcg1*) is reproduced in human hepatocytes [40]. Nevertheless, the identification of “Silent Cholesterol Disruption” as an incipient pathology of NAFLD represents a significant scientific advancement. Current drinking water guidelines are largely based on acute liver injury endpoints. Our findings suggest that these guidelines may need to be redefined to protect against chronic, sub-clinical metabolic toxicity. By ignoring this blind spot, we may be overlooking a significant environmental contributor to the global NAFLD pandemic.

### 3.13. Policy Recommendations: Closing the Regulatory Gap for Metabolic Toxicity

The results of this study—demonstrating that chronic low-dose exposure to microcystin-LR functions as a potent metabolic disruptor—signify a necessary expansion of the toxicological framework. Crucially, the dosage employed (10 µg/L) possesses direct public health relevance. Rather than an arbitrary experimental overdose, this concentration mirrors peak levels detected in finished drinking water during treatment compromises (e.g., the Lake Erie/Celina plant incidents) and in crops irrigated with contaminated sources [4,10].

#### 3.13.1. The “Blind Spot” in Current Guidelines

Our data highlight a critical regulatory blind spot. While the WHO provisional guideline (1 µg/L) is calibrated primarily to prevent acute hepatocellular injury [10], our findings demonstrate that exposure levels frequently encountered during bloom seasons trigger significant silent pathology (cholesterol accumulation) without inducing overt organ hypertrophy or systemic metabolic abnormalities. This implies that safety standards established for acute toxicity may fail to protect against chronic metabolic diseases like NAFLD. Furthermore, this risk is likely amplified in vulnerable populations. Studies using genetic NAFLD models (*Lepdb*/*J*) indicate that pre-existing metabolic conditions sensitize the liver to microcystin-LR, leading to exacerbated injury and mortality at doses well below the conventional NOAEL [15,16,17]. Therefore, environmental risk assessments must evolve to prioritize the protection of these “metabolically vulnerable” subpopulations rather than focusing solely on healthy averages.

#### 3.13.2. From Metabolic Toxicity to Tumor Promotion

The implication of this silent toxicity extends beyond metabolic disease. Classically, microcystin-LR is recognized as a tumor promoter [9]. The LDLR-driven cholesterol accumulation and subsequent oxidative stress identified here likely establish the chronic inflammatory microenvironment (e.g., NASH via the NOX2-miR-21 axis) that fuels this tumor-promoting potential [16,38]. Thus, preventing this early metabolic stage is crucial for blocking the progression to Hepatocellular Carcinoma (HCC) [27,37].

#### 3.13.3. Call for a Paradigm Shift

Consequently, we recommend a paradigm shift in environmental surveillance. Regulatory authorities should consider integrating molecular markers of flux disruption—specifically *Ldlr* and *Abcg1* mRNA profiles—as early biomarkers of toxicity. Moving beyond single-toxin evaluations to realistic models that account for mixture toxicity (co-exposure with high-fat diets or other chemicals [15,38]) is imperative. This comprehensive approach will enable the early detection of public health risks at the molecular level, facilitating preventive interventions before irreversible liver disease establishes [37,38].

## 4. Conclusions

Chronic ingestion of microcystin-LR may elevate the risk of progression to HCC mediated by NAFLD. Through molecular and multivariate analyses, this study strongly suggests that hepatic cholesterol accumulation serves as the primary trigger in the incipient stages of microcystin-LR-induced NAFLD, preceding systemic metabolic alterations. The molecular mechanism underlying this hepatic T-CHOL accumulation is characterized not by the upregulation of cholesterol synthesis, but by anomalies at the mRNA level in both influx and efflux pathways: specifically, a marked increase in *Ldlr* mRNA and a concurrent decrease in *Abcg1* mRNA. This specific mRNA profile supports the working hypothesis that microcystin-LR-induced PP2A inhibition induces *Ldlr* expression via post-transcriptional regulation (mRNA stabilization), bypassing the canonical SREBP-2 pathway. Given the lack of change in *Hmgcr* mRNA expression and the suggested functional suppression of synthetic activity due to PP2A inhibition, statins are unlikely to be effective therapeutic candidates for mitigating microcystin-LR-induced T-CHOL accumulation. Consequently, therapeutic strategies aimed at suppressing *Ldlr* expression or enhancing cholesterol efflux via *Abcg1* upregulation represent more promising approaches to arrest the progression of NAFLD caused by microcystin-LR exposure. Finally, this study proposes that in the environmental risk assessment of microcystin-LR, molecular alterations in *Ldlr* and *Abcg1* should be adopted as early biomarkers of metabolic toxicity, replacing the reliance solely on conventional acute toxicity indicators.

Our investigation suggests that chronic exposure to environmentally relevant 10 µg/L microcystin-LR triggers “Silent Hepatic Cholesterol Overload”—defining the first molecular step of environmental NAFLD. This study highlights three key findings:

1. Specific Phenotype: We identified a unique metabolic signature characterized by PLS-DA-confirmed liver T-CHOL accumulation (+16%, *p* = 0.01) that distinctively spares TAG metabolism (PLS1 vs. PLS2 segregation, Effect size: Cohen’s *d* = 1.596), separating environmental toxicity from diet-induced models.
2. Dual Failure Mechanism: The pathology is driven by a specific molecular collapse: Ldlr influx enhancement (LASSO coef +0.661) concurrent with an *Abcg1* efflux blockade (PLS1 loading −0.358), creating a potent cholesterol trap.
3. Regulatory Uncoupling: We resolved the paradox of *Srebp2* suppression despite *Ldlr* upregulation (Spearman ρ = −0.585, *p* = 0.0042) by elucidating a PP2A-mediated post-transcriptional override, suggesting that the toxin hijacks mRNA stability to bypass homeostatic feedback.

Clinical Implications: Our findings suggest that environmental NAFLD represents a distinct clinical entity characterized by pharmacological mismatch: since de novo synthesis is suppressed, this pathology is likely statin-refractory. Consequently, therapeutic strategies for toxicant-associated fatty liver disease (TAFLD) must shift toward targeting influx/efflux imbalances (e.g., ezetimibe or LXR agonists).

Policy Imperative: Finally, we posit that current WHO guidelines (1 µg/L)—focused primarily on acute hepatotoxicity—may not fully capture the risk of chronic metabolic disruption suggested by our findings. The integration of *Ldlr*/*Abcg1* biomarkers into environmental monitoring is essential to detect incipient toxicity and protect metabolically vulnerable populations from this silent epidemic.

## 5. Materials and Methods

### 5.1. Materials and Reagents

The following materials were obtained from the indicated suppliers: Microcystin-LR and aprotinin (FUJIFILM Wako Pure Chemical Corporation, Osaka, Japan); isoflurane (Pfizer Japan Inc., Tokyo, Japan); and heparin (Nacalai Tesque, Inc., Kyoto, Japan).

### 5.2. Animals and Experimental Design

All animal experiments and procedures were approved by the Animal Care and Use Committee of Shimane University (Protocol codes: MA28-1 and MA31-3). The experiments were conducted in accordance with the institutional regulations of Shimane University, the Act on Welfare and Management of Animals (Act No. 105), and relevant Japanese standards and guidelines. To eliminate potential variations in hormonal levels and organ function associated with the estrus cycle in females, male rats were selected for this study. Five-week-old wild-type male rats (WKAH/HkmSlc) were obtained from Japan SLC, Inc. (Hamamatsu, Japan). To minimize variability in food and water intake, rats were housed individually. However, to reduce isolation stress, transparent cages were placed in close proximity to allow visual contact between animals. Each cage was provided with corn cob bedding (Alpha-cob). Bedding was replaced weekly to maintain a low-stress environment. The animals were maintained in a climate-controlled room at 22 ± 2 °C with a relative humidity of 55 ± 5% under an automated 12 h light/dark cycle (light period: 08:00–20:00). Rats were given ad libitum access to an AIN-93G diet (free of the antioxidant *t*-butylhydroquinone to prevent interference with oxidative stress markers) and deionized water. Following a one-week acclimatization period, rats were randomly divided into two groups (*n* = 11 per group): a control group receiving deionized water and an experimental group receiving microcystin-LR dissolved in deionized water at a concentration of 10 μg/L for 7 weeks [5,23]. This specific concentration was selected to model environmentally relevant exposure rather than acute toxicity. It corresponds to the maximum levels detected in finished drinking water during treatment compromises (e.g., the Celina plant incident), as well as levels found in edible crops irrigated with contaminated water from Lake Erie and other sources [4,10]. Excessive weight loss due to microcystin-LR intake was established as a humane endpoint. The rats were monitored daily for clinical signs throughout the study. To minimize handling stress, food and water (or microcystin-LR-containing water) were replenished every 1–3 days, at which time body weight and intake were measured. To minimize potential confounding variables, plastic cages for each group were randomly arranged, and all measurements and dissections were performed in a randomized order. At the end of the treatment period, abdominal aortic blood was collected under anesthesia (induction with 5% isoflurane; maintenance with 2% isoflurane via nasal cannula) using a syringe containing heparin (final concentration: 50 U/mL blood) and aprotinin (final concentration: 500 kIU/mL blood). The collected blood was centrifuged at 2000× *g* for 10 min at 4 °C to obtain plasma. The plasma and harvested liver tissues were stored at −80 °C until analysis.

### 5.3. Measurement of Plasma Parameters and Liver Lipids

Plasma parameters and hepatic TAG and T-CHOL levels were measured using established methods as previously described in our reports [25,42]. Briefly, liver lipids were extracted and solubilized prior to quantification. The following commercial kits were used: NEFA C-Test Wako, Triglyceride E-Test Wako, Glucose CII-Test Wako, and Cholesterol E-Test Wako (FUJIFILM Wako Pure Chemical Corporation, Osaka, Japan).

### 5.4. Quantitative Real-Time PCR

Total RNA was extracted from homogenized rat liver tissues using the RNeasy Mini Kit (QIAGEN, Hilden, Germany). The mRNA expression levels of target genes were evaluated using PrimeScript™ RT Master Mix (Perfect Real Time) and TB Green™ Premix Ex Taq™ II (Tli RNaseH Plus) on a Thermal Cycler Dice Real Time System III (Takara Bio Inc., Shiga, Japan). These procedures were performed according to our established protocols [43,44,45]. The oligonucleotide primers used in this study are listed in Table 17. Primers were designed using NCBI Primer-BLAST (https://www.ncbi.nlm.nih.gov/tools/primer-blast/ (accessed on 3 December 2025)), targeting *Rattus norvegicus* RefSeq mRNA sequences. Candidate primers were selected with reference to orthologous mouse sequences from PrimerBank (https://pga.mgh.harvard.edu/primerbank/ (accessed on 1 December 2025)) to ensure coverage of conserved functional domains. Primer specificity was rigorously validated through in silico analysis with Primer-BLAST to exclude off-target amplification, followed by experimental confirmation via qPCR melt-curve analysis, which showed a single dissociation peak for each primer pair. Amplification products were quantified based on standard curves of known DNA concentrations, and quantification cycle (Cq) values were plotted against the log-transformed sample concentrations. The mRNA expression levels of each gene were normalized to that of ribosomal protein lateral stalk subunit P0 (*Rplp0*), which served as the internal control. This selection was based on a prior validation study comparing four common housekeeping genes (*Rplp0*, β-actin, glycerol-3-phosphate dehydrogenase, and cyclophilin), which identified *Rplp0* as the most reliable reference for gene expression analysis [46].

### 5.5. Statistical Analysis

#### 5.5.1. Univariate Analysis

All data are presented as mean ± standard error. Statistical significance was defined as *p* < 0.05. Comparisons of physiological and biochemical parameters, as well as the statistical comparison of PLS scores (PLS1 and PLS2) between the control and microcystin-LR groups (Figure 6B,C), were performed using Student’s *t*-test. These univariate analyses were conducted using Microsoft Excel for Mac, version 16.103.1 (Microsoft Corp., Redmond, WA, USA).

#### 5.5.2. Multivariate and Correlation Structure Analysis

Multivariate and correlation structure analyses were performed in a Python (version 3.12) environment. Prior to multivariate analysis, all continuous variables were standardized (mean = 0, standard deviation = 1) using the StandardScaler function in the scikit-learn library (version 1.4.2).

Partial Least Squares Discriminant Analysis (PLS-DA): To evaluate group separation based on overall metabolic profiles, PLS-DA models were constructed using the PLSRegression algorithm in scikit-learn. Two latent components (PLS1 and PLS2) were extracted. Group separation was visualized using score plots, and variable contributions were assessed using loading weights. Variable selection in PLS-DA was strictly based on Variable Importance in Projection (VIP) scores calculated according to the formula by Chong and Jun [47]. Consistent with established chemometric practice, variables with VIP > 1.0 were considered to have above-average importance and were retained for interpretation. No manual pre-selection of variables was applied prior to model construction. We did not use permutation testing because model performance was evaluated using ROC/AUC with 5-fold stratified cross-validation and bootstrap confidence intervals, which provide direct measures of predictive performance.

Receiver Operating Characteristic (ROC) and Bootstrap Analysis: To evaluate the discriminative performance of the PLS-DA model, ROC analysis was performed, and the Area Under the Curve (AUC) was computed using scikit-learn (metrics.roc_curve, metrics.auc, and metrics.roc_auc_score). Two classifiers were assessed: (i) a univariate classifier using the PLS1 score as the decision variable and (ii) a bivariate classifier combining PLS1 and PLS2 scores via logistic regression (solver = ‘liblinear’), with class probabilities used for ROC calculation. In addition to reporting full-dataset AUCs, five-fold stratified cross-validation was applied to estimate out-of-sample performance (fold-wise and mean AUCs). To quantify uncertainty for the full-dataset AUCs, 1000-iteration nonparametric stratified bootstrap resampling was used to derive 95% confidence intervals (CIs).

Multivariate ANOVA (MANOVA): To statistically validate the multidimensional group separation observed in the PLS-DA score space, Multivariate Analysis of Variance (MANOVA) was performed using the latent component scores (PLS1 and PLS2) as dependent variables. Test statistics (Wilks’ lambda, Pillai’s trace, Hotelling-Lawley trace, and Roy’s greatest root) were computed using the statsmodels library.

Feature Selection and Correlation Analysis: To quantitatively identify predictors of Liver T-CHOL, PLS1 scores, and PLS2 scores, Least Absolute Shrinkage and Selection Operator (LASSO) regression with cross-validation was applied using the LassoCV function in scikit-learn. The regularization parameter (α) was optimized via five-fold stratified cross-validation. Variables retaining non-zero coefficients in the final model were interpreted as important predictors. For binary classification (control vs. microcystin-LR) feature selection, L1-regularized logistic regression (LogisticRegressionCV; penalty = ‘l1’, solver = ‘liblinear’) was used, with the regularization parameter (*C*) optimized via five-fold stratified cross-validation. Spearman’s rank correlation coefficients (ρ) were calculated to evaluate relationships between variables (e.g., liver T-CHOL vs. *Ldlr*). Effect sizes (Cohen’s *d* and eta-squared (η^2^)) were computed for all between-group comparisons.

## Figures and Tables

**Table 1:** Effect of chronic low-dose microcystin-LR exposure on body and liver weights.

	Control	Microcystin-LR	*p*
Liver weight (g)	11.3 ± 0.64	11.5 ± 0.50	0.39
Liver weight (Liver-to-body weight ratio (%))	2.9 ± 0.08	2.9 ± 0.07	0.48

Data are presented as mean ± standard error (SE) (*n* = 11 per group). Statistical significance was evaluated using Student’s *t*-test. No significant differences were observed in absolute liver weight or liver-to-body weight ratio, confirming the absence of overt organ hypertrophy or cachexia at this dose (10 µg/L).

**Table 2:** Systemic metabolic parameters in plasma.

	Control	Microcystin-LR	*p*
Glucose (mg/dL)	233.6 ± 8.26	232.4 ± 5.25	0.45
Total cholesterol (mg/dL)	51.2 ± 1.81	51.8 ± 1.58	0.40
Triglyceride (mg/dL)	120.9 ± 18.75	131.8 ± 18.86	0.34
NEFA (mEq/dL)	1.09 ± 0.06	1.14 ± 0.04	0.26

Data are presented as mean ± SE (*n* = 11 per group). Statistical significance was evaluated using Student’s *t*-test. No significant differences were observed in glucose or lipid parameters, characterizing the “silent” nature of the toxicity in the systemic circulation. Abbreviations: NEFA, Non-Esterified Fatty Acids.

**Table 3:** Hepatic lipid profile showing specific cholesterol accumulation.

	Control	Microcystin-LR	*p*
Total cholesterol (mg/g liver)	8.6 ± 0.39	10.0 ± 0.41	0.01
Triglyceride (mg/g liver)	12.3 ± 0.96	12.7 ± 1.10	0.39

Data are presented as mean ± SE (*n* = 11 per group). Statistical significance was evaluated using Student’s *t*-test. Note the specific elevation of Total Cholesterol (+16%) in the absence of significant Triglyceride changes.

**Table 4:** Statistical evaluation of group separation on PLS axes.

Component	*t*	*p*	Cohen’s *d*	*η* ^2^
PLS1	3.568	0.0011	1.596	0.389
PLS2	1.567	0.0665	0.701	0.109

Effect size metrics (Cohen’s *d* and *η*^2^) were calculated to quantify the magnitude of separation between control and microcystin-LR groups along the first (PLS1) and second (PLS2) latent components. PLS1 demonstrates an extremely large effect size (*d* = 1.596), identifying it as the primary axis of toxicity. Note: Values represent the magnitude of the effect size. Higher scores in the microcystin-LR group indicate a positive shift relative to control. Abbreviations: PLS, partial least squares.

**Table 5:** Multivariate ANOVA (MANOVA) results.

Test Statistic	Value	Num DF	Den DF	*F*	*p*
Wilks’ lambda	0.50	2	19	9.44	0.0014
Pillai’s trace	0.50	2	19	9.44	0.0014
Hotelling-Lawley trace	0.99	2	19	9.44	0.0014
Roy’s greatest root	0.99	2	19	9.44	0.0014

MANOVA was performed using PLS1 and PLS2 scores as dependent variables to assess the overall difference between experimental groups. All four test statistics indicate a robust multidimensional group difference (*p* = 0.0014), confirming a distinct toxicological fingerprint. Abbreviations: DF, Degrees of Freedom.

**Table 6:** Loading weights of variables on the toxicity-specific PLS1 axis.

Variable	PLS1 Loading
Liver T-CHOL	+0.453
*Ldlr*	+0.377
*Cyp3a2*	+0.340
*Srebp1c*	+0.178
*Ppara*	+0.177
*Abcg5*	+0.141
Plasma NEFA	+0.137
*Pxr*	+0.136
*Abcg8*	+0.120
plasma TAG	+0.089
Liver TAG	+0.059
Plasma T-CHOL	+0.054
*Scarb1*	−0.020
Plasma glucose	−0.027
*Hmgcs1*	−0.073
*Hmgcr*	−0.083
*Rxr*	−0.129
*Abca1*	−0.156
*Pcsk9*	−0.222
*Srebp2*	−0.261
*Lxr*	−0.291
*Abcg1*	−0.358

Variables are ordered by the magnitude of their loading weights. Positive loadings indicate a positive association with the microcystin-LR induced phenotype (toxicity), while negative loadings indicate suppression. Note the opposing directions of *Ldlr* (positive) and *Srebp2* (negative), statistically corroborating the regulatory uncoupling hypothesis. Abbreviations: T-CHOL, Total Cholesterol; TAG, Triglycerides; NEFA, Non-Esterified Fatty Acids.

**Table 7:** Variable Importance in Projection (VIP) scores.

Variable	VIP Score	Note
Liver T-CHOL	1.879	Significant Driver
*Ldlr*	1.591	Significant Driver
*Abcg1*	1.505	Significant Driver
*Cyp3a2*	1.422	Significant Driver
*Srebp1c*	1.290	Significant Driver
*Lxr*	1.230	Significant Driver
*Srebp2*	1.170	Significant Driver
*Pcsk9*	1.133	Significant Driver
Plasma T-CHOL	0.762	
*Ppara*	0.761	
*Rxr*	0.752	
Liver TAG	0.749	
Plasma TAG	0.741	
*Hmgcr*	0.721	
*Abcg5*	0.716	
*Abca1*	0.657	
Plasma NEFA	0.616	
*Abcg8*	0.609	
*Pxr*	0.574	
*Scarb1*	0.541	
*Hmgcs1*	0.313	
Plasma glucose	0.174	

VIP scores identify the most influential variables in the PLS-DA model. Variables with VIP > 1 (e.g., Liver T-CHOL, *Ldlr*, *Abcg1*) are considered significant drivers of the separation between control and microcystin-LR groups.

**Table 8:** LASSO regression coefficients predicting the PLS1 toxicity score.

Variable	LASSO Coefficient
Liver T-CHOL	+0.485
*Cyp3a2*	+0.375
*Ldlr*	+0.354
*Srebp1c*	+0.208
*Abcg5*	+0.142
Liver TAG	+0.130
Plasma TAG	+0.129
Plasma NEFA	+0.106
*Ppara*	+0.092
*Pxr*	+0.089
Plasma T-CHOL	+0.020
*Hmgcs1*	−0.009
*Hmgcr*	−0.025
Plasma glucose	−0.044
*Pcsk9*	−0.226
*Abca1*	−0.240
*Lxr*	−0.383
*Srebp2*	−0.451

Least Absolute Shrinkage and Selection Operator (LASSO) regression was used to select sparse predictors of the PLS1 score. Positive coefficients indicate variables that drive the toxic phenotype (e.g., *Ldlr* and *Cyp3a2*), while negative coefficients indicate variables suppressed by toxicity (e.g., *Srebp2* and *Abcg1*). Abbreviations: LASSO, Least Absolute Shrinkage and Selection Operator.

**Table 9:** Spearman correlation analysis with the PLS1 score.

Variable	Spearman_ρ	*p*
*Ldlr*	+0.692	0.00035
Liver T-CHOL	+0.655	0.00092
*Cyp3a2*	+0.514	0.01431
*Abcg8*	+0.506	0.01615
*Abcg5*	+0.452	0.03456
*Abcg1*	−0.461	0.03069
*Lxr*	−0.526	0.01176
*Pcsk9*	−0.579	0.00467
*Srebp2*	−0.585	0.00419

Spearman’s rank correlation coefficients (ρ) assessing the monotonic relationship between individual variables and the toxicity axis (PLS1). Significant correlations (*p* < 0.05) confirm the variables’ association with the toxic phenotype.

**Table 10:** LASSO regression coefficients predicting Hepatic Total Cholesterol.

Variable	LASSO Coefficient
*Ldlr*	+0.661
Plasma glucose	+0.570
*Abcg8*	+0.321
*Hmgcr*	+0.263
*Cyp3a2*	+0.152
*Abca1*	−0.0965
*Hmgcs1*	−0.113
*Lxr*	−0.139
Plasma NEFA	−0.220
*Pcsk9*	−0.290
Plasma TAG	−0.431

LASSO regression identifying the molecular predictors of the primary phenotypic outcome (Liver T-CHOL). *Ldlr* is identified as the strongest positive predictor (+0.661), supporting the influx-driven accumulation hypothesis.

**Table 11:** Spearman correlation analysis with Hepatic Total Cholesterol.

Variable	Spearman_ρ	*p*
*Ldlr*	+0.535	0.01015
*Cyp3a2*	+0.426	0.04787

Spearman’s rank correlation analysis validating the association between gene expression and the phenotypic outcome (Liver T-CHOL). Significant positive correlations with *Ldlr* and *Cyp3a2* support their mechanistic roles in influx and compensatory metabolism, respectively.

**Table 12:** Loading weights of variables on the physiology-related PLS2 axis.

Variable	PLS2 Loading
*Srebp1c*	+0.527
Plasma T-CHOL	+0.512
Plasma TAG	+0.508
*Pcsk9*	+0.379
Liver TAG	+0.317
Plasma glucose	+0.280
*Hmgcs1*	+0.075
*Lxr*	+0.069
*Ldlr*	+0.044
*Srebp2*	+0.034
*Cyp3a2*	−0.059
*Hmgcr*	−0.0641
Liver T-CHOL	−0.118
*Abca1*	−0.132
Plasma NEFA	−0.135
*Scarb1*	−0.173
*Ppara*	−0.218
*Rxr*	−0.238
*Abcg5*	−0.240
*Pxr*	−0.256
*Abcg1*	−0.275
*Abcg8*	−0.297

Loading weights for the second latent component (PLS2). High loadings for *Srebp1c*, Plasma T-CHOL, and Plasma TAG indicate that this axis captures general physiological variations in lipid metabolism, independent of the microcystin-LR treatment effect.

**Table 13:** LASSO regression coefficients predicting the PLS2 score.

Variable	LASSO Coefficient
*Srebp1c*	+0.584
Plasma T-CHOL	+0.423
Liver TAG	+0.318
Plasma TAG	+0.281
*Hmgcr*	+0.262
*Pcsk9*	+0.202
Liver T-CHOL	+0.157
Plasma NEFA	+0.146
*Ldlr*	+0.034
*Cyp3a2*	+0.026
*Hmgcs1*	+0.009
Plasma glucose	−0.040
*Scarb1*	−0.074
*Abca1*	−0.141
*Abcg1*	−0.202
*Abcg5*	−0.223
*Rxr*	−0.318
*Lxr*	−0.331

LASSO regression identifying predictors of the PLS2 axis. *Srebp1c* is the strongest predictor, confirming this axis represents canonical lipogenic regulation.

**Table 14:** Spearman correlation analysis with the PLS2 score.

Variable	Spearman_ρ	*p*
Plasma TAG	+0.745332979	<0.0001
*Srebp1c*	+0.684923772	0.0004
Plasma T-CHOL	+0.679784166	0.0005
*Pcsk9*	+0.463579898	0.0297
Plasma glucose	+0.460712561	0.0309
Liver TAG	+0.4432524	0.0388
*Abcg8*	−0.425183512	0.0485
*Ppara*	−0.425183512	0.0485
*Pxr*	−0.473743648	0.0259
*Rxr*	−0.529079616	0.0113

Spearman’s rank correlation analysis confirming the strong association of PLS2 with markers of general lipid metabolism (Plasma TAG and *Srebp1c*), contrasting with the toxicity-specific profile of PLS1.

**Table 15:** LASSO regression coefficients predicting Plasma TAG.

Variable	LASSO Coefficient
*Srebp1c*	+28.028
*Pxr*	−0.284
Plasma NEFA	−2.061
Liver T-CHOL	−3.517
*Scarb1*	−15.038

LASSO regression identifying predictors of Plasma TAG levels. The exceptionally high coefficient for *Srebp1c* (+28.028) confirms that triglyceride synthesis remains under canonical transcriptional control, unlike the uncoupled cholesterol pathway.

**Table 16:** Spearman correlation analysis with Plasma TAG.

Variable	Spearman_ρ	*p*
*Srebp1c*	+0.673	0.0005
Plasma T-CHOL	+0.449	0.0356
*Ppara*	−0.428	0.0463
Plasma NEFA	−0.432	0.0443
*Pxr*	−0.551	0.0077
*Rxr*	−0.598	0.0032

Spearman’s rank correlation analysis confirming the canonical positive relationship between *Srebp1c* and Plasma TAG (*p* < 0.001), demonstrating intact lipogenic regulation.

**Table 17:** Forward (Fw) and reverse (Rv) primers for the target genes.

Target Genes	GenBank Accession No.	Primers (5→3′)	Length (bp)	Product Length (bp)
*Srebp2*	NM_001033694.2	Fw: TGGGCGATGAGCTGACTCT	19	80
		Rv: CAAGTCAGAGAACTCTCCCAC	21	
*Hmgcs1*	NM_017268.2	Fw: CGGATCGCGAAGACATCAACTC	22	88
		Rv: CGCCCAATGCAGTCATAGGAA	21	
*Hmgcr*	NM_013134.2	Fw: AGCTTGCCCGAATTGTGTGTG	21	104
		Rv: TCTGTTGTGAACCATGTGACTTC	23	
*Scarb1*	NM_031541.2	Fw: TTTGGAGTGGTAGTAAAAAGGGC	23	71
		Rv: TGACATCAGAGACTCAGAGTAG	22	
*Ldlr*	NM_175762.3	Fw: CTCCTGTATTCACGGTAGCC	20	124
		Rv: CCCACTGTGGCACTTGAATTTG	22	
*Pcsk9*	NM_199253.2	Fw: GCACTGGAGAACCACACAGG	20	102
		Rv: TGGCTGCATGACATTGCTTCTC	22	
*Abca1*	NM_178095.3	Fw: GCTTGTTGGTCTCAGTTAAGG	21	135
		Rv: GTAGCTCAGGCGGACAGAAAT	21	
*Abcg5*	NM_053754.2	Fw: AGAGGGCCTCACAACAACAGA	21	110
		Rv: CTGACGCTGAAGGACACATTC	21	
*Abcg8*	NM_130414.2	Fw: CTGTGGAACGGGACTGTACTCC	22	105
		Rv: GTTGGACTGACCACTGTAGGT	21	
*Abcg1*	NM_053502.2	Fw: GCTCCATCGTCTGCACCATCC	21	88
		Rv: ACACATTGTCCTTGACTTAGG	21	
*Cyp3a2*	NM_153312.3	Fw: GCTCTTGATGCATGGTTAAAGATTTG	26	99
		Rv: ATCACAGACCTTGCCAACTCCTT	23	
*Pxr*	NM_052980.2	Fw: CAAGAGCGACGGGAAAGAGAT	21	92
		Rv: CTTTGGCGAAGTTGATGACGC	21	
*Rxr*	NM_012805.3	Fw: ATGGACACCAAACATTTCCTGC	22	79
		Rv: CTCGACCCGTTGGAGAGCT	19	
*Lxr*	NM_031627.2	Fw: TCAGCATCTTCTCTGCAGACCGG	23	144
		Rv: TCATTAGCATCCGTGGGAACA	21	
*Srebp1c*	NM_001276707.1	Fw: TGACCCGACTATTCTGTG	18	61
		Rv: CTGGGCTGAGCGATACAGTTC	21	
*Ppara*	NM_013196.2	Fw: AACATCGAGTGTCGAATATGTGG	23	99
		Rv: CCGAATAGTTCGCCGAAAGAA	21	
*Rplp0*	NM_022402.2	Fw: GCTCCAAGCAGATGCAGCA	19	143
		Rv: CCGGATGTGAGGCAGCAG	18	

## Data Availability

The original contributions presented in this study are included in the article/Supplementary Materials. Further inquiries can be directed to the corresponding author.

## Supplementary Materials

The following supporting information can be downloaded at: https://www.mdpi.com/article/10.3390/toxins18020092/s1, Table S1: Five-fold stratified cross-validation (CV) results for the PLS-DA model.

## Abbreviations

The following abbreviations are used in this manuscript:

*Abca1*	ATP-binding cassette transporter A1
*Abcg1*	ATP-binding cassette transporter G1
*Abcg5*	ATP-binding cassette transporter G5
*Abcg8*	ATP-binding cassette transporter G8
AMPK	AMP-activated protein kinase
AUC	Area Under the Curve
CI	Confidence Interval
CyanoHABs	Cyanobacterial Harmful Algal Blooms
*Cyp3a2*	Cytochrome P450 3A2
*Hmgcr*	3-hydroxy-3-methylglutaryl-CoA reductase
*Hmgcs1*	3-hydroxy-3-methylglutaryl-CoA synthase 1
LASSO	Least Absolute Shrinkage and Selection Operator
*Ldlr*	Low-density lipoprotein receptor
*Lxr*	Liver X receptor (Nr1h3)
MANOVA	Multivariate Analysis of Variance
NAFLD	Non-alcoholic fatty liver disease
NEFA	Non-esterified fatty acids
OATPs	Organic anion transporting polypeptides
*Pcsk9*	Proprotein convertase subtilisin/kexin type 9
PLS-DA	Partial Least Squares Discriminant Analysis
*Ppara*	Peroxisome proliferator-activated receptor α
PP2A	Protein phosphatase 2A
*Pxr*	Pregnane X receptor (Nr1i2)
ROC	Receiver Operating Characteristic
*Rxr*	Retinoid X receptor
*Scarb1*	Scavenger receptor class B member 1
SE	Standard Error
*Srebp1c*	Sterol regulatory element-binding protein 1c
*Srebp2*	Sterol regulatory element-binding protein 2
TAG	Triglycerides
T-CHOL	Total Cholesterol
VIP	Variable Importance in Projection
WHO	World Health Organization

## References

[1] Yan X., Xu X., Wang M., Wang G., Wu S., Li Z., Sun H., Shi A., Yang Y. (2017). Climate warming and cyanobacteria blooms: Looks at their relationships from a new perspective. Water Res..

[2] Huisman J., Codd G.A., Paerl H.W., Ibelings B.W., Verspagen J.M.H., Visser P.M. (2018). Cyanobacterial blooms. Nat. Rev. Microbiol..

[3] Harke M.J., Steffen M.M., Gobler C.J., Otten T.G., Wilhelm S.W., Wood S.A., Paerl H.W. (2016). A review of the global ecology, genomics, and biogeography of the toxic cyanobacterium, *Microcystis* spp. Harmful Algae.

[4] Buratti F.M., Manganelli M., Vichi S., Stefanelli M., Scardala S., Testai E., Funari E. (2017). Cyanotoxins: Producing organisms, occurrence, toxicity, mechanism of action and human health toxicological risk evaluation. Arch. Toxicol..

[5] Koto Y., Kawahara H., Kurata K., Yoshikiyo K., Hashiguchi A., Okano K., Sugiura N., Shimizu K., Shimizu H. (2022). Microcystin-LR incorporated into colonic cells through probenecid-sensitive transporters leads to upregulated MCP-1 expression induced by JNK activation. Toxicol. Rep..

[6] Du X., Liu H., Yuan L., Wang Y., Ma Y., Wang R., Chen X., Losiewicz M.D., Guo H., Zhang H. (2019). The diversity of cyanobacterial toxins on structural characterization, distribution and identification: A systematic review. Toxins.

[7] Xing Y., Xu Y., Chen Y., Jeffrey P.D., Chao Y., Lin Z., Li Z., Strack S., Stock J.B., Shi Y. (2006). Structure of protein phosphatase 2A core enzyme bound to tumor-inducing toxins. Cell.

[8] MacKintosh C., Beattie K.A., Klumpp S., Cohen P., Codd G.A. (1990). Cyanobacterial microcystin-LR is a potent and specific inhibitor of protein phosphatases 1 and 2A from both mammals and higher plants. FEBS Lett..

[9] Nishiwaki-Matsushima R., Ohta T., Nishiwaki S., Suganuma M., Kohyama K., Ishikawa T., Carmichael W.W., Fujiki H. (1992). Liver tumor promotion by the cyanobacterial cyclic peptide toxin microcystin-LR. J. Cancer Res. Clin. Oncol..

[10] Miller T.R., Beversdorf L.J., Weirich C.A., Bartlett S.L. (2017). Cyanobacterial Toxins of the Laurentian Great Lakes, Their Toxicological Effects, and Numerical Limits in Drinking Water. Mar. Drugs.

[11] Zhang F., Lee J., Liang S., Shum C.K. (2015). Cyanobacteria blooms and non-alcoholic liver disease: Evidence from a county level ecological study in the United States. Environ. Health.

[12] Zhao Y., Yan Y., Xie L., Wang L., He Y., Wan X., Xue Q. (2020). Long-term environmental exposure to microcystins increases the risk of nonalcoholic fatty liver disease in humans: A combined fisher-based investigation and murine model study. Environ. Int..

[13] He J., Li G., Chen J., Lin J., Zeng C., Chen J., Deng J., Xie P. (2017). Prolonged exposure to low-dose microcystin induces nonalcoholic steatohepatitis in mice: A systems toxicology study. Arch. Toxicol..

[14] Li H., Yu X.H., Ou X., Ouyang X.P., Tang C.K. (2021). Hepatic cholesterol transport and its role in non-alcoholic fatty liver disease and atherosclerosis. Prog. Lipid Res..

[15] Lad A., Su R.C., Breidenbach J.D., Stemmer P.M., Carruthers N.J., Sanchez N.K., Khalaf F.K., Zhang S., Kleinhenz A.L., Dube P. (2019). Chronic Low Dose Oral Exposure to Microcystin-LR Exacerbates Hepatic Injury in a Murine Model of Non-Alcoholic Fatty Liver Disease. Toxins.

[16] Lad A., Hunyadi J., Connolly J., Breidenbach J.D., Khalaf F.K., Dube P., Zhang S., Kleinhenz A.L., Baliu-Rodriguez D., Isailovic D. (2022). Antioxidant Therapy Significantly Attenuates Hepatotoxicity following Low Dose Exposure to Microcystin-LR in a Murine Model of Diet-Induced Non-Alcoholic Fatty Liver Disease. Antioxidants.

[17] Arman T., Lynch K.D., Montonye M.L., Goedken M., Clarke J.D. (2019). Sub-Chronic Microcystin-LR Liver Toxicity in Preexisting Diet-Induced Nonalcoholic Steatohepatitis in Rats. Toxins.

[18] Li Z., Zheng D., Zhang T., Ruan S., Li N., Yu Y., Peng Y., Wang D. (2023). The roles of nuclear receptors in cholesterol metabolism and reverse cholesterol transport in nonalcoholic fatty liver disease. Hepatol. Commun..

[19] Rice L.M., Donigan M., Yang M., Liu W., Pandya D., Joseph B.K., Sodi V., Gearhart T.L., Yip J., Bouchard M. (2014). Protein Phosphatase 2A (PP2A) Regulates Low Density Lipoprotein Uptake through Regulating Sterol Response Element-binding Protein-2 (SREBP-2) DNA Binding. J. Biol. Chem..

[20] Adachi S., Homoto M., Tanaka R., Hioki Y., Murakami H., Suga H., Matsumoto M., Nakayama K.I., Hatta T., Iemura S. (2014). ZFP36L1 and ZFP36L2 control LDLR mRNA stability via the ERK-RSK pathway. Nucleic Acids Res..

[21] Abidi P., Zhou Y., Jiang J.D., Liu J. (2005). Extracellular signal-regulated kinase-dependent stabilization of hepatic low-density lipoprotein receptor mRNA by herbal medicine berberine. Arterioscler. Thromb. Vasc. Biol..

[22] Letourneux C., Rocher G., Porteu F. (2006). B56-containing PP2A dephosphorylate ERK and their activity is controlled by the early gene IEX-1 and ERK. EMBO J..

[23] Hitsuda Y., Koto Y., Kawahara H., Kurata K., Yoshikiyo K., Nishimura K., Hashiguchi A., Maseda H., Okano K., Sugiura N. (2024). Increased Prorenin Expression in the Kidneys May Be Involved in the Abnormal Renal Function Caused by Prolonged Environmental Exposure to Microcystin-LR. Toxics.

[24] Chen L., Hu Y., He J., Chen J., Giesy J.P., Xie P. (2017). Responses of the Proteome and Metabolome in Livers of Zebrafish Exposed Chronically to Environmentally Relevant Concentrations of Microcystin-LR. Environ. Sci. Technol..

[25] Lee J.Y., Shimizu H., Hagio M., Fukiya S., Watanabe M., Tanaka Y., Joe G.H., Iwaya H., Yoshitsugu R., Kikuchi K. (2020). 12α-Hydroxylated bile acid induces hepatic steatosis with dysbiosis in rats. Biochim. Biophys. Acta Mol. Cell Biol. Lipids.

[26] Yap F., Craddock L., Yang J. (2011). Mechanism of AMPK suppression of LXR-dependent Srebp-1c transcription. Int. J. Biol. Sci..

[27] Carmichael W.W. (1992). Cyanobacteria secondary metabolites—The cyanotoxins. J. Appl. Bacteriol..

[28] Moldavski O., Zushin P.H., Berdan C.A., Van Eijkeren R.J., Jiang X., Qian M., Ory D.S., Covey D.F., Nomura D.K., Stahl A. (2021). 4β-Hydroxycholesterol is a prolipogenic factor that promotes SREBP1c expression and activity through the liver X receptor. J. Lipid. Res..

[29] Joseph B.K., Liu H.Y., Francisco J., Pandya D., Donigan M., Gallo-Ebert C., Giordano C., Bata A., Nickels J.T. (2015). Inhibition of AMP Kinase by the Protein Phosphatase 2A Heterotrimer, PP2APpp2r2d. J. Biol. Chem..

[30] Samari H.R., Møller M.T., Holden L., Asmyhr T., Seglen P.O. (2005). Stimulation of hepatocytic AMP-activated protein kinase by okadaic acid and other autophagy-suppressive toxins. Biochem. J..

[31] Björkhem I. (2002). Do oxysterols control cholesterol homeostasis?. J. Clin. Investig..

[32] Woolsey S.J., Mansell S.E., Kim R.B., Tirona R.G., Beaton M.D. (2015). CYP3A Activity and Expression in Nonalcoholic Fatty Liver Disease. Drug Metab. Dispos..

[33] Fisher C.D., Lickteig A.J., Augustine L.M., Ranger-Moore J., Jackson J.P., Ferguson S.S., Cherrington N.J. (2009). Hepatic cytochrome P450 enzyme alterations in humans with progressive stages of nonalcoholic fatty liver disease. Drug Metab. Dispos..

[34] Clarke P.R., Hardie D.G. (1990). Regulation of HMG-CoA reductase: Identification of the site phosphorylated by the AMP-activated protein kinase in vitro and in intact rat liver. EMBO J..

[35] Ching Y.P., Kobayashi T., Tamura S., Hardie D.G. (1997). Specificity of different isoforms of protein phosphatase-2A and protein phosphatase-2C studied using site-directed mutagenesis of HMG-CoA reductase. FEBS Lett..

[36] Xian L., Hou S., Huang Z., Tang A., Shi P., Wang Q., Song A., Jiang S., Lin Z., Guo S. (2015). Liver-specific deletion of Ppp2cα enhances glucose metabolism and insulin sensitivity. Aging.

[37] Michelotti G.A., Machado M.V., Diehl A.M. (2013). NAFLD, NASH and liver cancer. Nat. Rev. Gastroenterol. Hepatol..

[38] Sarkar S., Alhasson F., Kimono D., Albadrani M., Seth R.K., Xiao S., Porter D.E., Scott G.I., Brooks B., Nagarkatti M. (2020). Microcystin exposure worsens nonalcoholic fatty liver disease associated ectopic glomerular toxicity via NOX-2-MIR21 axis. Environ. Toxicol. Pharmacol..

[39] Nishiwaki-Matsushima R., Nishiwaki S., Ohta T., Yoshizawa S., Suganuma M., Harada K.I., Suguri H., Fujiki H. (1994). Structure–function relationships of microcystins, liver tumor promoters, in interaction with protein phosphatase. Jpn. J. Cancer Res..

[40] Chowdhury R.R., Rose S., Ezan F., Sovadinová I., Babica P., Langouët S. (2024). Hepatotoxicity of cyanotoxin microcystin-LR in human: Insights into mechanisms of action in the 3D culture model Hepoid-HepaRG. Environ. Pollut..

[41] Niture S., Gadi S., Qi Q., Rios-Colon L., Khatiwada S., Vandana V., Fernando R.A., Levine K.E., Kumar D. (2023). Cyanotoxins Increase Cytotoxicity and Promote Nonalcoholic Fatty Liver Disease Progression by Enhancing Cell Steatosis. Toxins.

[42] Shimoda T., Shimizu H., Iwasaki W., Liu H., Kamo Y., Tada K., Hanai T., Hori S., Joe G.H., Tanaka Y. (2023). A diet supplemented with cholic acid elevates blood pressure accompanied by albuminuria in rats. Biosci. Biotechnol. Biochem..

[43] Tomii A., Higa M., Naito K., Kurata K., Kobayashi J., Takei C., Yuasa K., Koto Y., Shimizu H. (2023). Activation of the TLR4-JNK but not the TLR4-ERK pathway induced by indole-3-acetic acid exerts anti-proliferative effects on Caco-2 cells. Biosci. Biotechnol. Biochem..

[44] Ichisaka Y., Yano S., Nishimura K., Niwa T., Shimizu H. (2024). Indoxyl sulfate contributes to colorectal cancer cell proliferation and increased EGFR expression by activating AhR and Akt. Biomed. Res..

[45] Higa M., Naito K., Sato T., Tomii A., Hitsuda Y., Tahara M., Ishii K., Ichisaka Y., Sugiyama H., Kobayashi R. (2025). Hexaraphane Affects the Activation of Hepatic PPARα Signaling: Impact on Plasma Triglyceride Levels and Hepatic Senescence with Aging. Nutrients.

[46] Akamine R., Yamamoto T., Watanabe M., Yamazaki N., Kataoka M., Ishikawa M., Ooie T., Baba Y., Shinohara Y. (2007). Usefulness of the 5′ region of the cDNA encoding acidic ribosomal phosphoprotein P0 conserved among rats, mice, and humans as a standard probe for gene expression analysis in different tissues and animal species. J. Biochem. Biophys. Methods.

[47] Chong I.G., Jun C.H. (2005). Performance of some variable selection methods when multicollinearity is present. Chemom. Intell. Lab. Syst..

