# Supplementary material for: Microcystin-LR Drives Early NAFLD Pathogenesis via Hepatic Cholesterol Accumulation: Dysregulation of Ldlr and Abcg1 Expression Uncoupled from Srebp2
Source: Toxins (Basel). 2026 Feb 11;18(2):92. doi: 10.3390/toxins18020092 (PMC12945090; doi:10.3390/toxins18020092)
Supplement: Supplementary file 1 [file toxins-18-00092-s001.zip › toxins-4057632-supplementary.pdf]

Table S1. Five-fold stratified cross-validation (CV) results for the PLS-DA model. AUCs for the PLS1 classifier (univariate) and the combined classifier (PLS1+PLS2 via logistic regression) are shown for each fold, along with fold-specific sample composition. Mean ± SE summarizes performance across folds. See Methods for full CV settings; corresponding ROC curves are shown in Figure 6A.

Fold	N_test	Cont_test	MCLR_test	AUC (PLS1)	AUC (PLS1+PLS2)
**1**	5	3	2	0.500	0.667
**2**	5	2	3	0.500	1.000
**3**	4	2	2	0.500	0.500
**4**	4	2	2	0.250	0.750
**5**	4	2	2	1.000	0.500
**Mean**	4.4	2.2	2.2	0.550	0.683
**SE**	0.2	0.2	0.2	0.122	0.093

Notes: Values in fold rows are AUCs computed on each fold's test set. Mean ± SE summarizes across 5 folds (SE = SD/√5). CV = cross-validation; ROC = receiver operating characteristic; AUC = area under the curve; PLS-DA = partial least squares-discriminant analysis; Cont = Control; MC-LR = microcystin-LR.

CV protocol: StratifiedKFold (n_splits = 5, shuffle = True, random_state = 42). Class probabilities from logistic regression (solver = "liblinear") were used to compute ROC/AUC for the combined classifier. Bootstrap confidence intervals (CIs) for full-dataset AUCs are reported in the main text/Figure 6 (nonparametric stratified resampling, B = 1,000).
